# Book Reviews

**Published:** 1890-03

**Authors:** 


### Book Reviews.

---

MEDICAL AND SURGICAL MEMOIRS. By Joseph Jones, M. D.

We greet with pleasure the third volume of this work. Although impossible to do justice to such a work as this, in the space at our command, a careful examination of it has afforded us both pleasure and instruction.

Coming as it does from the pen of a southern born and bred physician of large experience, both in private practice and hospital work, it is especially interesting to the practitioners in the Southern States.

The work abounds in maps and charts, extensive statistical tables and colored engravings. In the first half of the volume are discussed contagious and infectious diseases, with measures for their prevention and arrest.

The importance of such investigation cannot be over-estimated, and the author's position as president of the Board of Health of the State of Louisiana, for a number of years, gave him unusual facilities for conducting such research and making practical application of his conclusions.

Preventable causes of diseases and deaths are fully set forth and discussed from a scientific and practical standpoint. The author devotes considerable space to the subject of yellow fever, and gives a detailed statement of the relations of heat and moisture to the origin and spread of the disease.

In the second half of the volume are discussed the scientific application of the philosophical principles of education in their bearing upon the medical profession. Some space is devoted to Teratology, or the science of monsters, giving the result of *post mortem* examinations upon some of the most noted monstrosities.

The author discusses advancement in the treatment of the insane during the nineteenth century, especially the treatment of the insane of Louisiana, public and international hygiene, progress of the discovery of disinfectants and their application for the arrest of contagion, and closes the volume with experiments testing the germicidal and antiseptic powers of substances employed externally and internally as germicides, and impresses the necessity for more careful and simpler experiments to determine the actual value of different antiseptics in the destruction of pathogenic organisms. L. H. J.

---

THE PRINCIPLES AND PRACTICE OF SURGERY. By John Ashhurst, Jr., M. D., Barton Professor of Surgery and Professor of Clinical Surgery in the University of Pennsylvania, Surgeon to Pennsylvania Hospital, etc., etc. Fifth edition enlarged and thoroughly revised, with six hundred and forty-two illustrations. Lea Bros. & Co., Philadelphia, 1889.

The rapid exhaustion of the previous editions of this excellent work is an attest of the esteem in which it is held by the profession.

The present edition shows careful revision and contains much that is new and valuable. Especially is this so of the chapter on "Antispetic Treatment of Wounds." The author has modified his views on the use of antiseptics as the result of personal experience and recommends their employment, still, however, in a rather tentative manner.

As a whole it is one of the best short treatises on surgery, and we take pleasure in heartily recommending it to the profession.

